# Childhood maltreatment and adult suicidality: a comprehensive systematic review with meta-analysis

**DOI:** 10.1017/S0033291718003823

**Published:** 2019-01-04

**Authors:** Ioannis Angelakis, Emma Louise Gillespie, Maria Panagioti

**Affiliations:** 1 School of Psychology, University of South Wales, Pontypridd, UK; 2 NIHR School for Primary Care Research, NIHR Greater Manchester Patient Safety Translational Research Centre, Manchester Academic Health Science Centre, University of Manchester, Manchester, UK

**Keywords:** Abuse, childhood maltreatment, meta-analysis, neglect, suicidality, systematic review

## Abstract

This comprehensive systematic review and meta-analysis aims to quantify the association between different types of childhood maltreatment and suicidality. We searched five bibliographic databases, including Medline, PsychINFO, Embase, Web of Science and CINAHL, until January 2018. Random-effects meta-analysis was employed followed by univariable and multivariable meta-regressions. Heterogeneity was quantified using the *I*^2^ statistic and formal publication bias tests were undertaken. The methodological quality of the studies was critically appraised and accounted in the meta-regression analyses. Data from 68 studies based on *n* = 261.660 adults were pooled. All different types of childhood maltreatment including sexual abuse [odds ratio (OR) 3.17, 95% confidence interval (CI) 2.76–3.64], physical abuse (OR 2.52, 95% CI 2.09–3.04) and emotional abuse (OR 2.49, 95% CI 1.64–3.77) were associated with two- to three-fold increased risk for suicide attempts. Similar results were found for the association between childhood maltreatment and suicidal ideation. Complex childhood abuse was associated with a particularly high risk for suicide attempts in adults (OR 5.18, 95% CI 2.52–10.63). Variations across the studies in terms of demographic and clinical characteristics of the participants and other core methodological factors did not affect the findings of the main analyses. We conclude that there is solid evidence that childhood maltreatment is associated with increased odds for suicidality in adults. The main outstanding challenge is to better understand the mechanisms which underpin the development of suicidality in people exposed to childhood maltreatment because current evidence is scarce.

It is estimated that up to 30% of the general population have experienced childhood maltreatment (Hussey *et al*., 2006). Physical, sexual and emotional or psychological abuse and neglect are among the most common types of maltreatment encountered by children and young people (Finkelhor *et al*., 2013). Experiences of childhood abuse and/or neglect precede the occurrence of psychiatric disorders in adult life, whereas 2.2% of incidents of childhood maltreatment result in fatalities (US Department of Health and Human Services, 2012). The economic burden of childhood maltreatment in terms of health care and medical costs, losses in productivity, welfare and special education costs are estimated around $124 billion in the USA alone (Fang *et al*., 2012).

Over 800 000 people across the world die by suicide every year. Understanding, therefore, the major factors which underpin suicidality such as suicide attempts, thoughts and behaviors has been established as a global health and policy priority [World Health Organization (WHO), 2014]. Empirical research has shown strong links between several types of childhood maltreatment and adult suicidality among individuals in the community and those diagnosed with psychiatric disorders (Gal *et al*., 2012; Kim *et al*., 2013). Consistent with the empirical findings, contemporary theories of suicidality have emphasized the role of childhood maltreatment, such as sexual and physical abuse, in the development of suicidality. For example, the interpersonal theory of suicide suggests that severe types of childhood maltreatment, such as sexual and/or physical abuse, produce a state of habituation to pain and reduction of fear for death which gradually builds the person's capability for suicide. Similarly, the Cry of pain model, and its antecessor, the Schematic Appraisals Model for Suicide (SAMS) suggest that childhood adversities give rise to increasingly worsening perceptions of defeat and entrapment which lead to suicidality as a means of escape (Johnson *et al*., 2008; Williams *et al*., 2005).

To date, two meta-analyses have confirmed the positive relationship between distinct types of childhood maltreatment and suicidality (Liu, *et al*., 2017; Zatti, *et al*., 2017). These meta-analyses, however, combined studies which were based on mixed samples of participants such as adolescents and adults, community and clinical samples. On the other hand, methodological restrictions have been applied regarding the definition of childhood maltreatment (e.g. use of the Childhood Trauma Questionnaire exclusively; CTQ; Liu *et al*., 2017), the study design (e.g. prospective studies only) and the date of publication (conducted within the last decade; Zatti *et al*., 2017), leading to the exclusion of several relevant studies. Moreover, little is known regarding the impact of demographic and clinical factors on the association between childhood maltreatment and suicidality. Although these studies are important, a comprehensive systematic review and meta-analysis would be particularly valuable for drawing important evidence-based conclusions and guiding future research priorities. We performed a comprehensive systematic review and meta-analysis of the association between childhood maltreatment and suicidality. We had two core objectives:

- To systematically quantify the association between different types of childhood maltreatment and suicidality, including suicide attempts and suicidal ideation.
- To examine demographic, clinical and methodological factors that may influence the association between childhood maltreatment and suicidality in adults.

## Methods

This systematic review and meta-analysis is aligned with the Preferred Reporting Items for Systematic Reviews and Meta-Analyses (PRISMA) statement (Moher *et al*., 2009) and the Meta-Analysis of Observational Studies in Epidemiology (MOOSE; Stroup *et al*., 2000).

### Eligibility criteria

Studies had to meet five criteria to be included in the review: (a) based on participants aged 18 years or older, who were exposed to childhood maltreatment such as abuse or neglect, (b) reported data on suicidality including suicide attempts, suicidal ideation or suicide deaths in adults exposed to childhood maltreatment (e.g. before the age of 18 years old) or reported a quantitative outcome of the association between childhood maltreatment and suicidality in adults; (c) focused on individuals from the community or individuals diagnosed with psychiatric disorders, (d) employed an observational quantitative research design and (e) written in English and published in peer-reviewed journals. We excluded studies which examined other types of maltreatment than abuse or neglect (e.g. bullying, parental divorce, loss/death of a loved one, separation from parents, witnessing violence), were based on veterans (this population differs from non-veterans on such variables as being pre-selected based on specific mental and physical criteria, having an increased likelihood of experiencing adversities in adulthood by encountering or witnessing stressful events and aging over 60 years old; U.S. Department of Veterans Affairs, 2014), and did not provide amendable data for meta-analyses.

### Search strategy and data sources

We searched five electronic bibliographic databases including Medline, PsychInfo, Embase, Web of Science and CINAHL. We also checked the reference lists of the identified studies to locate eligible studies and contacted the authors, whenever needed. The searches were conducted on 25 January 2018. Our search strategy included both text words and MeSH terms (Medical Subjective Headings) and combined two key blocks of key-terms: suicide (suicid* OR self*harm) and child/sexual/physical/emotional abuse or neglect or maltreatment or adversities (child*, sex*, phys*, emoti* abuse, negl*, maltreat*, advers*).

### Study selection

Two reviewers independently scrutinized the titles and the abstracts of the research papers identified. Then, the full texts of the potentially eligible studies were further evaluated independently by the two reviewers. Inter-rater reliability was high for title/abstract and full-text screening (*κ* = 0.95 and 0.97, respectively). Disagreements were resolved by discussions.

### Data extraction

First an electronic data extraction sheet was devised and piloted in six randomly selected studies. We extracted descriptive data on participant characteristics (e.g. age, gender), study characteristics (e.g. country, design, method of recruitment), screening tools for childhood maltreatment, type of maltreatment (e.g. sexual, physical and emotional/psychological abuse, and emotional or physical neglect), screening tools for suicidality and mode of suicidality (e.g. suicide attempts or suicidal ideation). Quantitative data for meta-analysis examining the association between childhood maltreatment and suicidality were also extracted. Two independent reviewers completed the data extraction. Inter-rater agreement was found to be as high as *κ* = 0.93 on 875 points checked.

### Critical appraisal assessment

The methodological quality of the included studies was critically appraised by using six criteria which were adapted by the CRD's guidance for undertaking reviews in health care (CRD, 2009) and the Quality Assessment Tool for Quantitative Studies (Thomas *et al*., 2004). These were: (i) research design (cross-sectional = 0, prospective/experimental = 1), (ii) baseline response rate (>70% or no reported = 0, <70% = 1), (iii) follow-up response rate (>70% or no reported = 0, <70% = 1), (iv) screening tools for childhood adversities (not reported/other = 0, structured/semi-structured clinical interview/self-report scale = 1), (v) screening tools for suicidality (not reported/other = 0, structured/semi-structured clinical interview/self-report scale = 1) and (vi) control for confounding/other factors in the analysis (no controlled/no reported = 0, controlled = 1). Studies which met at least four of these six criteria were considered to be of moderate to high quality, whereas studies which met fewer than four criteria were considered to be of low quality. Using this classification, a binary critical appraisal item was created across studies (1 = low quality appraisal score; 2 = moderate to high quality appraisal score) which was entered as moderator in the meta-regression analyses.

### Data analyses

Our primary outcome was the association between suicide attempts and different types of childhood maltreatment. All studies reported suicide attempts as dichotomous outcomes (number/proportions of participants with or without experiences of childhood maltreatment who engaged in suicide behavior in adulthood). Most studies (except for four studies) also reported suicidal ideation as dichotomous outcomes. Odds ratios (ORs) were, thus, selected as the preferred effect size across all analyses. Data from the four studies which reported secondary outcomes in different formats (e.g. mean score of suicidal ideation in participants with and without history of childhood maltreatment) were converted to ORs by utilizing a widely used formula (Borenstein *et al*., 2005). Most of the included studies contributed more than one relevant effect size for our analyses (e.g. the reported associations between several types of childhood maltreatment and suicide attempts). For this reason, we pooled the different types of childhood maltreatment separately to avoid double counting of studies in the same analysis.

We pooled all data in Stata 15 using the *metan* command and conducted univariable and multivariable meta-regressions to test the influence of study-level moderators on the associations between childhood maltreatment and suicide attempts using the *metareg* command (Harbord and Higgins, 2008). Six moderators were tested including mean age, percentage of men in the sample, population (community *v.* clinical sample), assessment method of suicide attempts and suicidal ideation (self-report questionnaire *v.* clinician or researcher administered interview), assessment method of childhood maltreatment (self-report questionnaire *v.* clinician or researcher administered interview) and critical appraisal score (low *v.* high score). We also examined whether the type of mental health condition (common *v.* severe) affected the relationship between childhood maltreatment and suicide attempts within the clinical sample. According to published guidance, each moderator value was based on a minimum of eight studies (Thompson and Higgins, 2002). Covariates meeting our significance criterion (*p* < 0.20) were entered into a multivariable meta-regression model. The *p* < 0.20 threshold was conservative, to avoid prematurely discounting potentially important explanatory variables.

Analyses were primarily conducted using a random effects model because we anticipated substantial heterogeneity, which was assessed with the *I*^2^ statistic (Higgins *et al*., 2003). For comparisons that included fewer than five effect sizes, we used a fixed effects model in cases of moderate to low heterogeneity (⩽50%). Conventionally, values of 25, 50 and 75% indicate low, moderate and high heterogeneity, respectively. Publication bias was examined by inspecting the funnel plots. Provided that the analyses were based on at least 9 studies, we applied formal tests including the Egger's tests (Egger *et al*., 1997). In case of possible publication bias, we used the Duval and Tweedie's trim-and-fill method, which corrects the estimated effect size by yielding an estimate of the number of the missing studies (Duval and Tweedie, 2000).

## Results

**Fig. 1.**
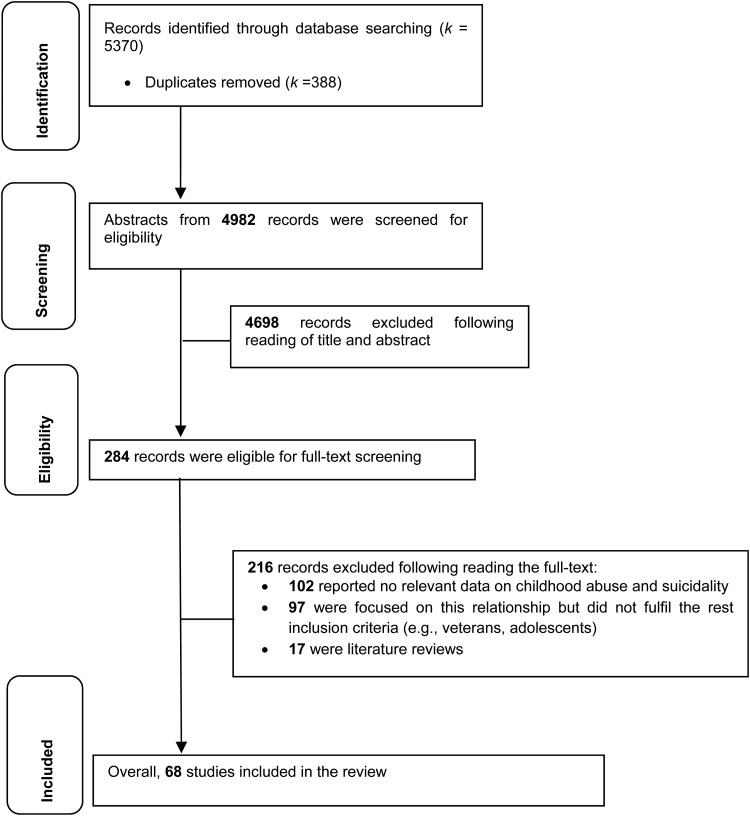
PRISMA 2009 flow diagram for the entire review.

A total of 5370 articles were retrieved. Of these, 388 were duplicates and 4698 were excluded because they (a) did not focus on suicidality, (b) focused on any other childhood maltreatment sub-types other than abuse or neglect (e.g. bullying, parental divorce, loss/death of a loved one, separation from parents, witnessing violence) and (c) were non-empirical studies, leaving 284 articles eligible for full-text screening. An additional 216 studies were excluded as they either did not report data relevant to the link between childhood abuse and suicidality or were based on adolescents or veterans. A total of 68 independent studies were included in the review (see Fig. 1).

### Descriptive characteristics of the studies

The characteristics of the 68 studies that were included in the review are detailed in Table 1. The vast majority of the studies were conducted in the United States (*k* = 29; 42.65%), followed by Canada (*k* = 7; 10.29%), Italy (*k* = 3; 4.41%), Turkey (*k* = 3; 4.41%), Germany (*k* = 3; 4.41%) and Brazil (*k* = 3; 4.41%). Fewer studies were conducted in the United Kingdom (*k* = 2; 2.94%), Australia (*k* = 2; 2.94%), New Zealand (*k* = 2; 2.94%), France (*k* = 2; 2.94%), Netherlands (*k* = 2; 2.94%), Poland (*k* = 2; 2.94%) and Korea (*k* = 2; 2.94%), whereas a single study (1.47%) was conducted in Argentina, Spain, Norway, Israel, Japan and South Africa. Stein *et al*. (2010) presented data that have been collected from 21 countries.

**Table 1.** Descriptive characteristics of the studies included in the review

Study	Country	Study design	Screening tool for abuse	Mode of childhood abuse	Screening tool for suicidality	Mode of suicidality	Target population	Sample size *N*	Men (%)	Age (years)	Q.A.
Afifi *et al*. (2008)	United States	Cross-sectional	The National Comorbidity Survey, conducted via face-to-face interviews involving three questions pertaining to childhood sexual and physical abuse	Childhood physical and sexual abuse	The National Comorbidity Survey, conducted via face-to-face interviews	Suicidal ideation and suicide attempts	General population	5692, response rate = 70.9%	n/r	18 years and above	4/5
Álvarez *et al*. (2011)	Spain	Cross-sectional	The Traumatic Life Events Questionnaire (TLEQ)	Childhood physical, sexual and psychological abuse	Study intake interview: asked the number of suicide attempts during their live	Suicide attempts	Schizophrenia, bipolar disorder or schizoaffective disorder	102, response rate = 100%	53% male	*M*_age_ = 39.4, s.d. = 10.4	4/5
Anderson *et al*. (2002)	United States	Cross-sectional	Three subscales of the Childhood Trauma Questionnaire (CTQ)	Childhood physical, sexual and emotional abuse	Evaluated by the principal investigator to determine the eligibility of the suicide attempt	Suicide attempts	Self-identified African-American women presenting for medical care	392, response rate = 91.84%	100% female	*M*_age_ = 32, s.d. = 10.38, range = 18–64	4/5
Andover *et al*. (2007)	United States	Cross-sectional	Clinician Administered Physical and Sexual Abuse Interview for Adults	Childhood physical and sexual abuse	Interview not specified	Suicide attempts	Participants with major depressive disorder	121, response rate = 76.86%	26.9% male	Range: 18–65	2/5
Beristianos *et al*. (2016)	United States	Cross-sectional	The World Health Organization's World Mental Health (WMH) survey	Childhood maltreatment	Assessed in its own section of the WMH-CIDI	Suicidal ideation	General population	20 013, response rate = 74.38%	47.9% male	*M*_age_ = 45	4/5
Bornovalova *et al*. (2011)	United States	Cross-sectional	Emotional, physical, and sexual abuse subscales of the Childhood Trauma Questionnaire-Short Form (CTQ-SF)	Childhood physical, sexual and emotional abuse	Items from the Self-Harm Inventory (SHI)	Suicidal attempts	Substance misuse inpatients	182, response rate = 98.90%	72% male	*M*_age_ = 43.05, s.d. = 9.86	4/5
Boudewyn and Liem, (1995)	United States	Cross-sectional	The Life Experience Survey designed for use in this study	Childhood sexual abuse	Questions about their experience of suicidal ideation, and suicide attempts	Suicidal ideation and suicide attempts	College students	438, response rate = 100%	39.5% male	*M*_age_ = 24.87	4/5
Brezo *et al*. (2008)	Canada	Prospective	The Childhood Sexual Abuse Scale and 14 questions selected from the revised conflict tactics scales (CTS2)	Childhood physical and sexual abuse	The Scale for Suicide Ideation and the Suicidal Intent Scale	Suicidal ideation and suicide attempts	College students	3017, response rate – 66%	52.83% male	*M*_age_ at final follow-up = 21.4	4/5
Briere and Runtz, (1986)	Canada	Cross-sectional	Data from patients’ intake interview	Childhood sexual abuse	Data from intake interview in the form of self-report variables	Suicidal ideation and suicide attempts	Women presenting at the Crisis Counseling program	195, response rate = 100%	100% female	*M*_age_ = 27.4	3/5
Brodbeck *et al*. (2018)	Germany	Cross-sectional	Childhood Trauma Questionnaire-Short Form (CTQ-SF)	Childhood abuse and neglect	The German version of the SCL-90-R	Suicidal ideation and suicide attempts	Outpatients recruited for a randomized trial	311, response rate = 77.36%	n/r	*M*_age_ = 40.4, s.d. = 10.58	4/5
Brodsky *et al*. (2001)	United States	Cross-sectional	The Columbia Demographic and Treatment History Interview	Childhood physical and sexual abuse	The Columbia Suicide History Form and the Suicide Intent Scale	Suicide attempts	Participants with major depressive episode	136, response rate = 100%	36.03% male	*M*_age_ = 39.5, s.d. = 12.9	3/5
Brown *et al*. (1999)	United States	Prospective	Obtained from New York State Central Registry for Child Abuse and Neglect (NYSCR)	Childhood maltreatment	National Institute of Mental Health Diagnostic Interview Schedule for Children (DISC)	Suicide attempts	General population	1141, rate at baseline = 85.54%, follow-up response rate = 56%	52.27% male	18 years old at fourth assessment	5/6
Bruwer *et al*. (2014)	South Africa	Cross-sectional	A modified version of the Conflict Tactics Scale (CTS2) and a standardized definition of rape followed by two questions: Did this ever happen to you?’ and ‘Other than rape, were you ever sexually assaulted or molested?’	Childhood physical and sexual abuse	V.3 of the WHO Composite International Diagnostic Interview (WHO CIDI)	Suicidal ideation and suicide attempts	General population	5089, response rate = 85.5%	46.3% male	*M*_age_ = 37	4/5
Bryan *et al*. (2013)	United States	Cross-sectional	Presented with a list of potentially stressful or traumatic events and asked to indicate whether they had ever experienced each	Childhood physical and sexual abuse	Beck Scale for Suicidal Ideation	Suicidal ideation and suicide attempts	College students	309, response rate = 100%	62.89% male	*M*_age_ = 19.83, s.d. = 3.15	3/5
Bryant and Range (1997)	United States	Cross-sectional	Child Sexual Abuse Questionnaire (CSAQ) and the Child Abuse Questionnaire	Childhood physical and sexual abuse	The Scale for Suicide Ideation, the Suicidal Behaviors Questionnaire, and the Brief Reasons for Living Inventory	Suicidal ideation	College students	486, response rate = 100%	26% male	*M*_age_ = 23.6	3/5
Carlier *et al*. (2016)	Netherlands	Cross-sectional	Childhood Trauma Questionnaire (CTQ)	Childhood physical, sexual, emotional abuse and emotional neglect	The suicidality section of the MINI- Plus	Suicide risk	Psychiatric outpatients	1245, response rate = 100%	39.6% male	*M*_age_ = 38.1, s.d. = 13.2	4/5
Daray *et al*. (2016)	Argentina	Cross-sectional	A semi-structure interview and dichotomously coded as yes or no	Childhood sexual abuse	Identified during initial evaluation	Suicide attempts	Participants admitted for care	177, Response rate = 100%	100% female	*M*_age_ = 37.6, s.d. = 11.68	3/5
de Mattos Sousa *et al*. (2016)	Brazil	Cross-sectional	Childhood Trauma Questionnaire (CTQ)	Childhood physical, sexual, emotional abuse and physical and emotional neglect	Mini International Neuropsychiatric Interview Plus version (MINI Plus)	Suicidal ideation, suicide plans and suicide attempts	Participants with major depressive disorder	473, response rate = 100%	16.3% male	*M*_age_ = 36.2, s.d. = 11.4, range: 18–60	4/5
Dervic *et al*. (2006)	United States	Cross-sectional	Columbia Demographic and Treatment History Interview	Childhood physical and sexual abuse	Columbia Suicide History Form	Suicidal ideation and suicide attempts	Participants with major depressive disorder	119, response rate = 100%	25.2% male	*M*_age_ = 36.8, s.d. = 11.5	4/5
Draper *et al*. (2008)	Australia	Cross-sectional	Two questions in a postal questionnaire survey: ‘Were you the victim of any physical abuse before you were 15 years old?’ and ‘Were you the victim of any sexual abuse before you were 15 years old?’	Childhood physical and sexual abuse	One question in a postal questionnaire survey: ‘How often have you attempted to kill yourself in your lifetime?’	Suicide attempts	Participants with major depressive disorder	77 820, response rate = <30%	41.3% male	*M*_age_ = 71.9, s.d. = 7.7	3/5
Dube *et al*. (2001)	United States	Cross-sectional, retrospective	Adverse Childhood Experiences (ACE) Study questionnaire and the Conflict Tactics Scale (CTS)	Childhood physical, sexual and emotional abuse	One question: ‘Have you ever attempted to commit suicide?’	Suicide attempts	General population (attending primary care)	26 824, response rate = 64.63%	54% male	*M*_age_ = 56, s.d. = 15.2	3/5
Easton *et al*. (2013)	United States	Cross-sectional	Three questions: ‘About how many times were you sexually abused by the abuser?’, ‘Did the abuser use physical force?’ and ‘Were you physically abused by someone close to you?’	Childhood physical and sexual abuse	Suicide attempts were measured using one item from the General Mental Health Distress Scale (GMDS). Suicidal ideation was measured using one item from the GMDS	Suicidal ideation and suicide attempts	Men with histories of CSA	487, response rate N/R, <70%	100% male	*M*_age_ = 50.37, s.d. = 10.82	4/5
Enns *et al*. (2006)	Netherlands	Prospective	Questions: ‘Before you reached the age of 16, were you ever psychologically abused? – physically abused? – sexually abused?’	Childhood physical, sexual, psychological abuse and childhood neglect	Respondents were asked: ‘Have you ever felt so low you thought about committing suicide?’ and ‘Have you ever attempted suicide?’	Suicidal ideation and suicide attempts	General population	7076, response rate baseline = 79. 4%, response rate at final follow-up = 47. 8%	n/r	Range = 18–64	5/6
Felitti *et al*. (1998)	United States	Cross-sectional	Questions from published surveys were used to construct the ACE Study questionnaire including the Conflicts Tactics Scale (CTS) and Wyatt (1985)	Childhood physical, sexual and psychological abuse	Behavioral Risk Factor Surveys, study questionnaire and the Health Appraisal Clinic's questionnaire	Suicide attempts	General population	13 494, response rate = 70.5%	48% male	*M*_age_ = 56.1	4/5
Fergusson *et al*. (1996)	New Zealand	Prospective	A private structured interview	Childhood sexual abuse	A private structured Interview	Suicide attempts	General population	1265, response rate at final follow-up = 79.13%	n/r	Studied from birth to 25 years old	4/5
Fudalej *et al*. (2015)	Poland	Cross-sectional	Unspecified questionnaire	Childhood sexual abuse	Suicidality Module of the Mini International Neuropsychiatric Interview (MINI) and the Beck Suicide Intent Scale (BSI)	Suicidal ideation and suicide attempts	Opioid dependent individuals	240, response rate = 100%	70.4% male	Median = 34, range = 18–55	2/5
Fuller-Thomson *et al*. (2012)	Canada	Cross-sectional	One question: ‘Were you ever physically abused by someone close to you?’	Childhood physical abuse	One question: ‘Have you ever seriously considered committing suicide or taking your own life?’	Suicidal ideation	General population	38 492, response rate = 17.3%	43.8% male	18 years and above	3/5
Fuller-Thomson *et al*. (2016)	Canada	Cross-sectional	One question: ‘How many times did an adult force you or attempt to force you into any unwanted sexual activity, by threatening you, holding you down or hurting you in some way?’	Childhood physical and sexual abuse	One question: ‘Have you ever attempted suicide or tried to take your own life?’	Suicide attempts	General population	43 030, response rate = 52.43%	49% male	*M*_age_ = 46.9, s.d. = 17.5	3/5
Gal *et al*. (2012)	Israel	Cross-sectional	Embedded into a section of the Israel National Health Survey (INHS) that assessed early and adult adverse experiences	Childhood physical and sexual abuse	INHS-version of the Composite International Diagnostic Interview (CIDI)	Suicidal ideation, suicide plans and suicide attempts	Jewish-Israeli respondents	6656, response rate = 57.88%	49.15% male	n/r	3/5
Garno *et al*. (2005)	United States	Cross-sectional	Childhood Trauma Questionnaire (CTQ)	Childhood physical, sexual, emotional abuse and physical and emotional neglect	Semi-structured interview	Suicide attempts	Patients with bipolar disorder	100, response rate = 100%	51% male	*M*_age_ = 41.6, s.d. = 12.3	4/5
Gould *et al*. (1994)	United States	Cross-sectional	An abuse questionnaire ascertaining information regarding six levels of unwanted physical contact	Childhood physical, sexual and emotional abuse	A history of suicide attempts was defined by an affirmative answer to the question, ‘Have you ever made a suicide attempt?’	Suicide attempts	General population (attending primary care)	778, response rate = 37.53%	28.77% male	*M*_age_ (female) = 40, *M*_age_ (male) = 42	3/5
Güleç *et al*. (2014)	Turkey	Cross-sectional	Childhood Trauma Questionnaire (CTQ)	Childhood physical, sexual, emotional abuse and physical and emotional neglect	A sociodemographic and clinical data form	Suicide attempts	Patients with conversion disorder	100, response rate = 100%	15% male	Range: 18–64	4/5
Gutierrez *et al*. (2000)	United States	Cross-sectional	The Childhood Sexual Experiences Questionnaire (CSEQ) and the Childhood Physical Experience Questionnaire (CPEQ)	Childhood sexual and physical abuse	The Multi-Attitude Suicide Tendency Scale (MAST) and the Adult Suicidal Ideation Questionnaire (ASIQ)	Suicidal ideation	College women	710, response rate = 90.7%	100% female	*M*_age_ = 18.59, s.d. = 1.08	3/5
Hardt *et al*. (2008)	Germany	Cross-sectional	Sections of the Mainz Structured Biographical Interview (MSBI)	Childhood physical and childhood sexual abuse	The Mainz Structured Biographical Interview (MSBI)	Suicide attempts	Psychiatric patients	575, response rate = 100%	25.22% male	*M*_age_ = 44.7, s.d. = 11.6	3/5
Hassan *et al*. (2016)	Canada	Cross-sectional	Childhood Trauma Questionnaire (CTQ)	Childhood physical, sexual, emotional abuse and physical and emotional neglect	Beck Scale for Suicidal Ideation (BSS), the Columbia-Suicide Severity Rating Scale (C-SSRS)	Suicide attempts	Patients with schizophrenia	361, response rate = 100%	67.87% male	*M*_age_ (no trauma) = 39.47, s.d. = 13.1, *M*_age_ (with trauma) = 44.63, s.d. = 12.38	4/5
Jakubczyk *et al*. (2014)	Poland	Cross-sectional	Substance Abuse Outcomes Module (SAOM)	Childhood sexual abuse	MINI International Neuropsychiatric Interview	Suicide attempts	Alcohol-dependent individuals	404, response rate = 95.54%	73.6% male	*M*_age_ = 43.4, s.d. = 9.9	4/5
Janiri *et al*. (2015)	Italy	Cross-sectional	Childhood Trauma Questionnaire (CTQ)	Childhood physical, sexual, emotional abuse and physical and emotional neglect	A semi-structured questionnaire consisting of two parts, one related to the past 6 months and the other to the lifetime (to the entire life)	Suicidal ideation and suicide attempts	Outpatients diagnosed with bipolar disorder (BD) I and bipolar disorder II	104, response rate = 100%	59.62% male	*M*_age_ (BD-I) = 43.93, *M*_age_ (BD-II) = 46.32	4/5
Jeon *et al*. (2014)	South Korea	Cross-sectional	The Early Trauma Inventory Self Report-Short Form (ETISR-SF)	Childhood physical, sexual and emotional abuse	Korean version of the Mini-International Neuropsychiatric Interview's (MINI) suicidality module	Suicidal ideation, suicide plans and suicide attempts	General population	2500, response rate = 54.76%	44% male	18 years and above	3/5
Joiner *et al*. (2007)	United States	Cross-sectional	The National Comorbidity Survey	Childhood physical abuse, molestation, rape and verbal abuse	Participants were asked whether they had ever attempted suicide, and if so, how many times	Suicidal attempts	General population	9804, response rate = 59.45%	50% male	*M*_age_ = 33.2, s.d. = 10.7	2/5
Kaplan *et al*. (1995)	United States	Cross-sectional	The Traumatic Experiences Questionnaire (TEQ)	Childhood physical and sexual abuse	Harkavy-Asnis Suicide Survey	Suicidal ideation, suicide plans and suicide attempts	Psychiatric outpatients	251, response rate = 100%	27% male	*M*_age_ = 38.7, range: 18–87	3/5
Kim *et al*. (2013)	Korea	Prospective	A checklist which allowed respondents to indicate events.	Childhood physical and sexual abuse	A semi-structured clinical interview and the Beck Scale for Suicide Ideation	Suicidal ideation and suicide attempts	Patients with a depressive disorder	1183, response rate = 74.22%	26.77% male	*M*_age_ = 46.1, s.d. = 15.9	4/5
Laglaoui-Bakhiyi *et al*. (2017)	France	Cross-sectional	Childhood Trauma Questionnaire (CTQ)	Childhood physical, sexual, emotional abuse and emotional neglect	Interview carried out by clinicians	Suicide ideation	Psychiatric patients with a history of suicide attempts	338, response rate = 100%	35%	*M*_age_ = 42.5 s.d. = 12.30	4/5
Leeners *et al*. (2014)	Germany	Cross-sectional	Interviews performed at support centers were used to diagnose	Childhood physical and sexual abuse	Investigated using yes/no answers	Suicidal ideation	Females from the general population	375, response rate = 68%	100% female	n/r	2/5
Lopez-Castroman *et al*. (2012)	France	Cross-sectional	The short version of the Childhood Trauma Questionnaire (CTQ)	Childhood physical, sexual, emotional abuse and physical and emotional neglect	Columbia Suicide History Form and the section O of the DIGS	Suicidal ideation, suicidal attempts and suicidal intent	Participants who attempted suicide	1563, response rate = 56.17%	29%	Range = 18–75	3/5
Marshall *et al*. (2013)		Prospective	Childhood Trauma Questionnaire (CTQ)	Childhood physical, sexual, emotional abuse and physical and emotional neglect	Two questions: ‘In the last 6 months, have you attempted suicide?’ and ‘Have you ever attempted suicide in your lifetime’	Suicide attempts	Illicit drug users	1634, response rate = 66.64%	67.9% male	Median = 42, range: 19–71	3/5
Martin *et al*. (2016)	Canada	Cross-sectional	Childhood Experiences of Violence Questionnaire (CEVQ)	Childhood sexual and physical abuse	Three questions from the Composite International Diagnostic Interview (CIDI)	Suicidal ideation, suicide plans and suicide attempts	General population	23 846, response rate = 3.47%	n/r	18 years and above	3/5
Martins *et al*. (2014)	Brazil	Cross-sectional	Childhood Trauma Questionnaire (CTQ)	Childhood physical, sexual, emotional abuse and physical and emotional neglect	Beck Scale for Suicide Ideation (BSI)	Suicidal ideation	Psychiatric patients	81, response rate = 100%	27% male	*M*_age_ = 37.62, s.d. = 1.21	3/5
McKenna and Gillen (2016)	N. Ireland	Cross-sectional	A modified version of Conflict Tactics Scale (CTS)	Sexual, physical abuse and neglect	Suicidality module of the WMH CIDI	Suicidality	General population	1986 response rate = 45.76%	47.83% male	Range: 18–93	3/5
Mert *et al*. (2015)	Turkey	Cross-sectional	Childhood Trauma Questionnaire (CTQ)	Childhood Physical, sexual, emotional abuse and physical and emotional neglect	A form designed by the researchers was used to collect sociodemographic variables and clinical variables	Suicide attempts	Outpatients with bipolar disorder	91, response rate = 100%	56% male	*M*_age_ = 38.3, s.d. = 11.7	4/5
Obikane *et al*. (2018)	Japan	Cross-sectional	Two questions: ‘Have you been physically abused by one or both of your parents before you finished a middle school?’ and ‘Have you experienced neglect by one or both of your parents before you finished a middle school?’	Childhood abuse, including physical abuse and neglect	World Mental Health Composite International Diagnostic Interview (CIDI)	Lifetime suicidal ideation, plans and attempts	General population	3792, response rate = 45.1%	46.85% male	Range = 20–50	3/5
Pérez-Fuentes *et al*. (2013)	United States	Cross-sectional	Questions adapted from the Adverse Childhood Event study, including questions from the Conflict Tactics Scale (CTS) and the Childhood Trauma Questionnaire (CTQ)	Childhood sexual abuse	The National Epidemiologic Survey on Alcohol and Related Conditions (NESARC) survey	Suicidal ideation and suicide attempts	General population	34 653, response rate = 98.74%	47.79% male	18 years and above	4/5
Perich *et al*. (2014)	Australia	Cross-sectional	An interview schedule	Childhood physical and sexual abuse	A clinical psychiatric symptom interview schedule	Suicidal attempts	Bipolar patients	201, response rate = 78.11%	36%	*M*_age_ = 38.55, s.d. = 13.43	3/5
Pompili *et al*. (2014)	Italy	Cross-sectional	The Childhood Trauma Questionnaire (CTQ)	Childhood physical, sexual, emotional abuse and physical and emotional neglect	The Suicidal History Self-Rating Screening Scale (SHSS)	Suicidal ideation, suicide plans and suicide attempts	Psychiatric patients	163, response rate = 86%	49.08% male	*M*_age_ = 42.1, s.d. = 14.2, range = 18 to 77	3/5
Read *et al*. (2001)	New Zealand	Cross-sectional	Files of clients whom were treated at a Community Mental Health Center	Childhood physical and sexual abuse	Client files	Suicidal ideation and suicide attempts	Psychiatric patients	200, response rate = 100%	43% male	*M*_age_ = 36.6, s.d. = 12.32, range = 18 to 69	3/5
Rossow and Lauritzen (2001)	Norway	Cross-sectional	Structured interview questionnaires with a question relating to childhood sexual abuse or violence	Childhood sexual abuse and violence	Structured interview questionnaire; included ‘Have you ever on purpose tried to take your own life?’ and a question about ideation	Suicidal ideation and suicide attempts	Residential and outpatient drug addicts	2391, response rate = 33.46%	100% male	n/r	3/5
Roy *et al*. (2007)	United States	Cross-sectional	Childhood Trauma Questionnaire (CTQ)	Childhood physical, sexual, emotional abuse and physical and emotional neglect	A semi-structured psychiatric interview conducted by a psychiatrist, collected socio-demographic variables, psychiatric history and history of attempting suicide	Suicide attempts	Abstinent substance-dependent patients	306, response rate = 83.99%	100% male	18 years and above	4/5
Roy and Janal (2006)	United States	Cross-sectional	Childhood Trauma Questionnaire (CTQ)	Childhood physical, sexual, emotional abuse and physical and emotional neglect	A semi-structured clinical psychiatric interview; Standardized definition of suicide attempts included	Suicide attempts	Abstinence, substance-dependent patients	1889, response rate = 100%	85.55% male	*M*_age_ = 40.3, s.d. = 8.0	4/5
Sadeh and McNiel (2013)	United States	Prospective	One questions: ‘Did anyone ever sexually abuse or assault you?’	Childhood sexual victimization	Questions regarding attempts with an intention of killing oneself	Suicidal attempts	Psychiatric patients	1136, response rate = 65.84%	56%	*M*_age_ = 30.0, s.d. = 6.23	3/5
Saraçlı *et al*. (2015)	Turkey	Cross-sectional	Childhood Trauma Questionnaire (CTQ)	Childhood physical, sexual, emotional abuse and physical and emotional neglect	Suicide Probability Scale (SPS)	Suicidal ideation and suicide attempts	General population	899, response rate = 99.11%	47.6% male	*M*_age_ = 39.4, s.d. = 12.4	4/5
Sarchiapone *et al*. (2007)	Italy	Cross-sectional	Childhood Trauma Questionnaire (CTQ)	Childhood physical, sexual, emotional abuse and physical and emotional neglect	A structured psychiatric interview	Suicide attempts	Patients with unipolar depression	108, response rate = 100%	38.89% male	8 years and above	4/5
Sfoggia *et al*. (2008)	Brazil	Cross-sectional	Childhood Trauma Questionnaire (CTQ)	Childhood physical, sexual, emotional abuse and physical and emotional neglect	Suicidal Behaviors Module of the WMH-CIDI	Suicidal ideation and suicide attempts	General population	125, response rate = 96%	40% male	*M*_age_ = 42.5, s.d. = 15.6	4/5
Shinozaki *et al*. (2013a)	United States	Cross-sectional	Obtained from electronic records based on the self-report of the patients during their thorough intake interview	Childhood physical, sexual, and emotional abuse	Electronic medical records of inpatient treatments	Suicidal ideation and suicide attempts	Patients with depressive episodes	250, response rate = 100%	30.5% male	*M*_age_ = 43, s.d. = 13	4/5
Shinozaki *et al*. (2013b)	United States	Cross-sectional	Obtained from electronic medical records of their inpatient treatment	Childhood physical, sexual and emotional abuse	Electronic medical records of inpatient treatments	Suicidal ideation and suicide attempts	Patients with depressive episodes	422, response rate = 100%	30.33% male	*M*_age_ = 44.27, s.d. = 12.9	4/5
Stansfeld *et al*. (2016)	United Kingdom	Prospective	A retrospective report at 45 years of age	Childhood physical and sexual abuse	The Revised Clinical Interview Schedule (CIS-R) and a single item from this scale: ‘In the past week have you felt that life is not worth living?’	Suicidal ideation	General population	17 416, response rate = 53.84%	49.72% male	45 years and above	4/5
Stein *et al*. (2010)	21 countries	Cross-sectional	The World Mental Health version of the World Health Organization Composite International Diagnostic Interview (CID)	Childhood adversities	Suicidality Module of the WMH-CIDI	Suicidal ideation and suicide attempts	Individuals from 21 different countries	102 245, response rate = 71.9%	n/r	18 years and above	4/5
Talbot *et al*. (2004)	United States	Cross-sectional	A single-item question administered during structured interview and by chart reviews	Childhood sexual abuse	Scale of Suicidal Ideation (SSI), and patients’ self-report and chart reviews	Death ideation, suicidal ideation and suicidal attempts	Psychiatric patients	127, response rate = 100%	100% female	50 years and above	4/5
Thompson *et al*. (2000)	United States	Cross-sectional	Childhood Trauma Questionnaire (CTQ)	Childhood physical, sexual, emotional abuse and physical and emotional neglect	Any woman who was release from hospital following a suicide attempt and women who presented in hospital for non-medical reasons	Suicide attempts	Women from the general population	335, response rate = 100%	100% female	*M*_age_ = 32.17, s.d. = 10.30	4/5
Twomey *et al*. (2000)	United States	Cross-sectional	Childhood Trauma Questionnaire (CTQ)	Childhood physical, sexual, emotional abuse and physical and emotional neglect	Recruited based on being at hospital after a serious attempt which resulted in a self-injurious act that required medical attention	Suicidal attempt	Participants admitted after suicidal attempt	159, response rate = 100%	100% female	Range = 18–64	3/5

*M*, mean; s.d., standard deviation; n/r, not reported; QA, quality appraisal of the methodology of the included studies.

The age of the participants ranged between 18 and 93 years old (*M*_age_ = 40.26, s.d. = 8.69; 42.34% men). In total, 33 studies (*n* = 225.462) were based on community samples and 35 on clinical samples (*n* = 36.198). Twenty-one of the studies that utilized psychiatric patients focused on common types of mental health conditions, including anxiety, depression, post-traumatic stress disorder (PTSD) and 14 on severe types of mental health conditions, such as bipolar disorder and schizophrenia.

Childhood maltreatment was assessed using two main methods: (i) 44 studies used self-report questionnaires and (ii) 23 studies used clinical interviews and other objective methods (e.g. five studies retrieved relevant information by patient records; only one study by Fudalej *et al*. (2015) did not specify the tools utilized). The most common self-report measure was the CTQ. Similarly, suicide attempts and suicidal thoughts were assessed either using self-report questionnaires (*k* = 33 studies) or structured/semi-structured clinical interviews or other objective methods (*k* = 35, of which five studies retrieved relevant information by patient records).

**Fig. 2.**
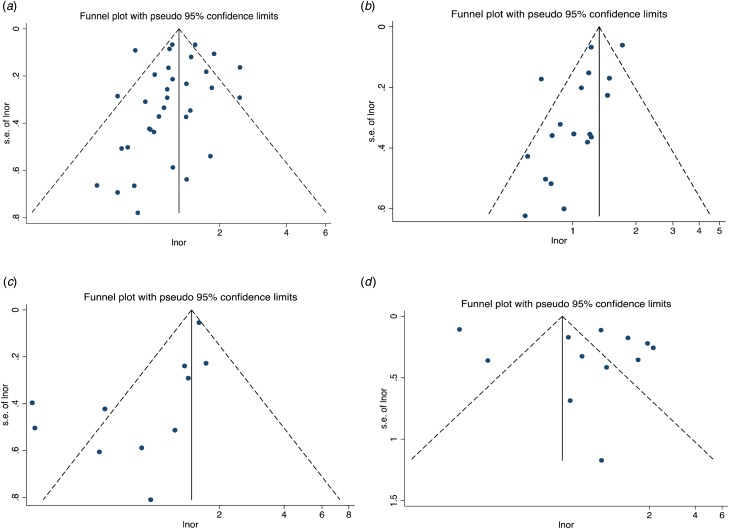
Funnel plots for effect sizes. Childhood maltreatment and suicide attempts: (*a*) sexual abuse, (*b*) physical abuse, (*c*) emotional abuse and (*d*) any child abuse.

Table 1 also presents the overall scores from the critical appraisal assessment of the studies. Almost half of the studies (*k* = 37) scored moderate to high in the critical appraisal assessment (met four or more criteria) whereas the remaining scored low (met fewer than four criteria).

### Main meta-analyses: associations between types of childhood maltreatment and suicide attempts in adults

The pooled effects of the main analyses indicate that all types of childhood maltreatment (except for physical neglect which is only based on four studies) were associated with significantly increased odds for suicide attempts in adults (Table 2). Sexual abuse was associated with a three-fold increased risk for suicide attempts [*k* = 36, OR 3.17, 95% confidence interval (CI) 2.76–3.64, *I*^2^ = 68.1%] whereas physical and emotional abuse were associated with a 2.5-fold increased risk for suicide attempts (*k* = 30, OR 2.52, 95% CI 2.09–3.04, *I*^2^ = 74.3% and *k* = 12, OR 2.49, 95% CI 1.64–3.77, *I*^2^ = 93.2%, respectively). Emotional neglect was associated with 2.3-fold increased risk for suicide attempts whereas physical neglect was not significantly associated with an increased risk for suicide attempts (*k* = 6, OR 2.29, 95% CI 1.79–2.94, *I*^2^ = 19.2% and *k* = 4, OR 1.51, 95% CI 0.87–2.62, *I*^2^ = 62.3%, respectively). However, the two categories focused on neglect were based on a small number of studies which have distinguished emotional/physical abuse from emotional/physical neglect. Moreover, a considerable number of studies examined the association between a combined category of childhood abuse (without providing data on each separate form of abuse) and suicide attempts. We named this combined category as *any child abuse* and analyzed it separately because it contains unspecified features from more than one of the other categories. Any child abuse was associated with a two-fold increased risk for suicide attempts (*k* = 16, OR 2.09, 95% CI 1.67–2.60, *I*^2^ = 91.3%). Finally, complex abuse (repetitive incidents) in childhood showed the strongest association (increased the risk five times) with suicide attempts among adults (*k* = 7, OR 5.18, 95% CI 2.52–10.63, *I*^2^ = 90.9%). As indicated by the value of *I*^2^ statistic, heterogeneity was medium to high across all the main analyses.

**Table 2.** Results of meta-analyses of the association between child maltreatment and suicidality (*k* = 68)

			Effect size analyses			Heterogeneity	Publication bias
	Total *k*	Total *N*	OR	95% CI	*p* value	*I*^2^ (%)	Trim-and-fill OR (95% CI)
Suicide attempts							
Sexual abuse	36^a^	210.763	3.17	2.76–3.64	<0.001	68.1	–
Physical abuse	24^a^	109.627	2.52	2.09–3.04	<0.001	74.3	–
Emotional abuse	12^a^	33.857	2.49	1.64–3.77	<0.001	93.2	2.36 (1.69–3.02)
Any child abuse	16	14.574	2.09	1.67–2.60	<0.001	91.3	–
Emotional neglect	6	1.777	2.29	1.79–2.94	<0.001	19.2	–
Physical neglect	4	955	1.51	0.87–2.62	0.14	62.3	–
Complex abuse	7	101.929	5.18	2.52–10.63	<0.001	90.9	–
Additional analyses							
Suicide ideation							
Sexual abuse	12^a^	112.626	2.15	1.77–2.62	<0.001	53.8	–
Physical abuse	11	32.083	2.43	1.85–3.18	<0.001	64.6	–
Emotional abuse	5^a^	5.936	2.10	1.51–2.94	<0.001	54.4	–
Any child abuse	7^a^	21.201	2.66	1.93–3.68	<0.001	81.4	–
Emotional neglect	4	2.176	1.40	1.02–1.93	0.04	80.3	–
Physical neglect	4	2.176	1.44	1.06–1.95	0.02	70.3	–

*k*, number of independent effect sizes; OR, pooled odds ratio, *N*, number of participants.

a One outlier was dropped from the analyses.

### Secondary meta-analyses: associations between types of childhood maltreatment and suicidal ideation in adults

All types of childhood maltreatment were associated with significantly increased odds for suicidal ideation in adults. Sexual abuse and emotional abuse were associated with a two-fold increased risk for suicidal ideation (*k* = 12, OR 2.15, 95% CI 1.77–2.62, *I*^2^ = 53.8% and *k* = 5, OR 2.10, 95% CI 1.51–2.94, *I*^2^ = 54.4%, respectively) whereas physical and any child abuse were associated with a 2.5-fold increased risk for suicidal ideation (*k* = 11, OR 2.43, 95% CI 1.85–3.18, *I*^2^ = 64.6% and *k* = 7, OR 2.66, 95% CI 1.93–3.68, *I*^2^ = 81.4%, respectively). Emotional and physical neglect were associated with 1.5-fold increased risk for suicidal ideation (*k* = 4, OR 1.40, 95% CI 1.02–1.93, *I*^2^ = 80.3% and *k* = 4, OR 1.44, 95% CI 1.06–1.95, *I*^2^ = 70.3%, respectively). As indicated by the value of *I*^2^ statistic, heterogeneity ranged from medium to high across all analyses.

### Small study bias

We assessed for publication bias across analyses which included at least nine studies (see also funnel plots; Fig. 2). In none of the main (sexual abuse-suicide attempts, *p* = 0.28, physical abuse-suicide attempts *p* = 0.11, any child abuse-suicide attempts *p* = 0.06) and secondary analyses (sexual abuse-suicidal ideation, *p* = 0.08, physical abuse-suicidal ideation, *p* = 0.07), the Egger's test for publication bias was significant except for the association between emotional abuse and suicide attempts (bias = 0.57, 95% CI 0.14–1.00, *p* = 0.02). For this comparison we run the Duval and Tweedie's trim-and-fill method which reduced slightly the effect size.

### Meta-regressions exploring the variance in the association between childhood maltreatment and suicide attempts

The results of the univariable and multivariable analyses are shown in Table 3. The numbers of pooled studies only allowed meta-regressions to examine factors which affect the associations between sexual abuse, physical abuse and suicide attempts.

**Table 3.** Meta-regression analyses

	Univariate moderator analyses				Multivariate meta-regression analyses			
	*b* (95% CI)	OR (95% CI)	*p* value	*I*^2^ (%)	*b* (95% CI)	*p* value	*I*^2^ (%)	*R*^2^ (%)
Suicide attempts								
Sexual abuse							41.02	36.97
Age (mean)	0.02 (−0.01 to 0.06)	–	0.09	49.87	0.02 (−0.01 to 0.06)	0.20		
Male gender %	−0.01 (−0.03 to 0.01)	–	0.44	57.80	–	–		
Population (Community/Clinical)	–	1.67 (0.77–3.64)	0.19	58.81	0.69 (−0.02 to 1.42)	0.06		
CM measure (Interview/scale)	–	1.66 (0.75–3.68)	0.21	54.58	–	–		
Suicidality measure (Interview/scale)	–	2.12 (0.99–4.57)	0.05	47.76	0.53 (−0.33 to 1.38)	0.22		
Critical appraisal score (Low/high)	–	0.97 (0.43–2.17)	0.94	58.29	–	–		
Type of mental condition (common/severe)^a^	–	1.09 (0.26–4.47)	0.90	64.22	–	–		
Physical abuse				24.16			51.87	44.53
Age (mean)	0.05 (0.01–0.09)	–	0.02	58.36	0.05 (0.02–0.08)	0.03		
Male gender %	−0.02 (−0.04 to 0.01)	–	0.10	68.58	−0.02 (−0.04 to 0.00)	0.07		
Population (Community/Clinical)		1.04 (0.39–2.81)	0.94	70.95	–	–		
CM measure (Interview/scale)	–	1.14 (0.37–3.51)	0.81	72.28	–	–		
Suicidality measure (Interview/scale)	–	0.93 (0.34–2.51)	0.87	71.77	–	–		
Critical appraisal score (Low/high)	–	0.61 (0.23–1.59)	0.30	68.20	−0.45 (−1.21 to 0.27)	0.26		
Type of mental condition (common/severe)^a^	–	0.49 (0.09–2.72)	0.38	79.61	–	–		

CM, childhood maltreatment.

a These analyses were based on the clinical sample only (*k* = 17 and *k* = 13 for the associations between sexual abuse and suicide attempts, and between physical abuse and suicide attempts, respectively).

#### Sexual abuse and suicide attempts (*k* = 36)

Studies which were based on older participants (*b* = 0.02, 95% CI −0.01 to 0.06, *p* = 0.09), community samples (OR 1.67, 95% CI 0.77–3.64, *p* = 0.19), and assessed suicide attempts using clinical interviews (OR 2.12, 95% CI 0.99–4.57, *p* = 0.05) tended to report a larger association between sexual abuse and suicide attempts in univariable analyses and were eligible for inclusion in multivariable analysis. The overall multivariable model was statistically significant χ^2^(3) = 3.11, *p* = 0.04 and reduced the *I*^2^ statistic from 68.1% to 41.02%. Population was the only predictor which approached significance in the multivariable analyses suggesting that the association of sexual abuse and suicide attempts may be stronger in community samples compared with clinical samples (*b* = 0.69, 95% CI −0.02 to 1.42, *p* = 0.06).

#### Physical abuse and suicide attempts (*k* = 25)

Studies based on older participants (*b* = 0.05, 95% CI 0.01–0.09, *p* = 0.02) and a higher percentage of women (*b* = −0.02, 95% CI −0.04 to 0.01, *p* = 0.10) tended to report a larger (although non-significant) association between physical abuse and suicide attempts in univariable analyses and were eligible for inclusion in multivariable analysis. The overall multivariable model was statistically significant χ^2^(3) = 3.99, *p* = 0.02 and reduced the *I*^2^ statistic from 74.3% to 51.87%. Age was significant predictor and gender approached significance in the multivariable analyses suggesting that the association between physical abuse and suicide attempts is stronger in studies based on older participants and women (*b* = 0.05, 95% CI 0.02–0.09, *p* = 0.03 and *b* = −0.02, 95% CI −0.04 to 0.00, *p* = 0.07).

Last, we examined whether common types of mental health conditions, including anxiety, depression and PTSD, or more severe conditions, such as bipolar disorder and schizophrenia, affected the association between childhood maltreatment and suicidality within clinical populations. However, our analyses did not support such a distinction (see Table 3).

## Discussion

### Summary of main findings

The most comprehensive systematic review and meta-analysis to date demonstrated that suicidality is a major concern in adults who have experienced core types of childhood maltreatment (e.g. abuse, neglect). A two- to three-fold increased risk for suicide attempts and suicidal ideation was identified in adults who experienced sexual, physical or emotional abuse as children compared with adults who have not experienced maltreatment during childhood. Adults exposed to sexual abuse and complex abuse in childhood were particularly vulnerable to suicidality. The relationship between major types of childhood maltreatment such as sexual and physical abuse and suicide attempts was not moderated by the characteristics of participants across studies (e.g. gender, the presence and severity of psychiatric diagnoses) and methodological variations (measures of childhood maltreatment or suicidality, critical appraisal scores). The only notable exception was that age moderated the association between physical abuse and suicide attempts, in that older participants were at higher risk for suicide attempts. The association between sexual abuse and suicide attempts also tended to be higher in community samples but this moderating effect was not significant. Our findings are especially supportive of early interventions to reduce suicide risk in people exposed to childhood maltreatment (as suicide risk could become more severe as these people age) and regular assessments for experiences of childhood maltreatment among people who self-harm or report thoughts of suicide followed by appropriate therapeutic management. Moreover, these findings encourage community interventions particularly for non-clinical populations who experience a significant but often untreated risk for suicide because these people are less likely to be in regular contact with mental health support services. In the latest case, community programs might have the most realistic potential to achieve intervention reach and be implementable at scale.

### Key research considerations and theoretical implications

The findings of the main analyses are generally consistent with the findings of less extensive systematic reviews in that childhood maltreatment is associated with a greater risk for suicide attempts (Liu *et al*., 2017; Zatti *et al*., 2017). We have advanced the existing literature with the use of a larger pool of studies which allowed us to further examine the impact of core moderators on the relationship between major types of childhood abuse and suicide attempts. Our methodology also enabled us to confirm a significant relationship between childhood abuse and suicidal ideation. Previous reviews reported non-significant pooled effect sizes of the association between childhood abuse and suicidal ideation but they were based on a considerably smaller number of studies compared with our analyses. In terms of suicide deaths, there is a paucity of evidence. We only identified one large retrospective study that presented relevant data on suicide deaths among 12 million participants (Cutajar *et al*., 2010); between 1964 and 1995 individuals who had been sexually abused had an 18-fold greater risk for dying by suicide (OR 18.14, 95% CI 9.05–36.36).

Older age was the only significant moderator of the association between physical abuse and suicide attempts. We speculate that as people who have been maltreated in childhood age, they are less capable of buffering the impact of life stresses or negative life events and therefore they gradually become less resilient and more prone to suicidality. More research is needed to investigate this hypothesis. Moreover, our finding that the relationship between childhood maltreatment and suicide attempts does not differ across people with severe mental health conditions, common mental health conditions and people from the community with no known mental health problems is of major importance because it suggests that the impact of childhood abuse on suicide risk is direct and trans-diagnostic. The finding that gender did not moderate the association between childhood maltreatment and suicide attempts seems to be consistent with the extant literature which suggests that males and females do not differ in their exposure to the overall number of adversities in childhood but rather in their exposure to specific types of childhood maltreatment (Freedman *et al*., 2002). For example, several studies have demonstrated that males experience more frequently physical abuse, whereas females encounter more frequently experiences of sexual abuse (e.g. Kessler *et al*., 1995).

In terms of theoretical implications, the findings of this review could suggest that all types of childhood maltreatment operate on suicidality via a single mechanism. Gibson and Leitenberg (2001) proposed that powerlessness mediates the relationship between sexual abuse and disengagement, defined as any form of cognitive or behavioral avoidance. Considering the commonalities between feelings of powerlessness and hopelessness, as well as defeat and entrapment, such appraisals could potentially mediate or moderate the relationship between childhood maltreatment and suicidality (e.g. O'Connor *et al*., 2011). However, there are no empirical findings to confirm this hypothesis. Furthermore, childhood abuse has also been found to be strongly associated with a diagnosis of PTSD which is one of the most well-known risk factors for suicidality (Davidson *et al*., 1991; Panagioti *et al*., 2009, 2012; Tarrier and Gregg, 2004; Taylor *et al*., 2011). Empirical research examining the mechanisms of suicidality in PTSD has supported the validity of contemporary models of suicidality, including the SAMS, which postulates that feelings of hopelessness (which often interact with recent negative experiences) lead to defeat and entrapment and subsequently to suicidality in PTSD patients (Johnson *et al*., 2008). A recent meta-analysis also highlighted the trans-diagnostic sequel of defeat and entrapment which is present in several conditions including depression, anxiety, PTSD and suicidality (Siddaway *et al*., 2015). Thus, the mediating/moderating effects of such cognitive appraisals (including disengagement) remain to be tested in models aimed at identifying the underlying mechanisms leading to suicide for those who experienced childhood maltreatment.

### Strengths and limitations

This large systematic review and meta-analysis has several strengths. It has been conducted in accordance with published guidance (PRISMA and MOOSE guidelines) which entails the involvement of two independent researchers throughout the screening, data extraction and data synthesis, the critical appraisal of the methodological quality of the studies and the performance of inter-rater reliability tests and advanced statistical analyses (e.g. multivariable meta-regressions).

However, there are a number of limitations which warrant discussion. First, significant heterogeneity was detected across most of the analyses. We dealt with heterogeneity by applying random effect models and conducted multivariable meta-regression analyses to further explore key sources of heterogeneity among the studies. Second, the findings of this systematic review and meta-analysis have been derived by English language peer-reviewed papers. It is reassuring that formal tests indicated no evidence of publication bias in our core analyses. One exception was the analysis which examined the association between emotional abuse and suicide attempts in which publication bias was a potential threat. Although the pooled effect has been adjusted using the trim-and-fill approach, the results of this analysis should be interpreted with caution. Third, our core analyses included a relatively large number of studies which also allowed us to perform advanced analyses (e.g. meta-regressions). However, this option was not always appropriate because some analyses were based on a small number of studies (e.g. analyses of the associations between suicide attempts, suicidal ideation and emotional/physical neglect). Fourth, the majority of the studies in our analyses were cross-sectional. Although the occurrence of suicide attempts or suicidal ideation cannot temporally precede childhood abuse, large longitudinal studies are recommended to examine the causal mechanisms of suicidality among survivors of childhood abuse over time. Last, in this review, we have only considered core and severe types of childhood maltreatment. There is evidence that other types of childhood maltreatment such as experiences of bullying, separation from parents or dysfunctional attachment styles are linked with suicidality particularly in people with severe mental health conditions such as psychoses, bipolar disorder and borderline personality disorder. Another systematic review to examine the association between suicidality and experiences of bullying, separation and attachment styles is fruitful avenue for future research.

## Conclusion

This is the most comprehensive systematic review with meta-analysis to corroborate the relationship between childhood maltreatment and adult suicidality. Our main findings demonstrated that all types of childhood abuse are associated with increased risk for suicide attempts and suicidal ideation in adults independent of demographic, clinical and methodological variations across the studies. Beyond this, there is a major gap in the literature regarding the mechanisms by which experiences of childhood maltreatment exert their detrimental, long-lasting impact on suicide risk. A better understanding of the causal mechanisms of suicidality in people exposed to childhood maltreatment has the potential to guide the development of more efficient interventions which will specifically target these causal mechanisms.

## Supplementary material

For supplementary material accompanying this paper visit https://doi.org/10.1017/S0033291718003823. click here to view supplementary material
