# Supplementary material for: Childhood maltreatment and adult suicidality: a comprehensive systematic review with meta-analysis
Source: Psychol Med. 2019 Jan 4;49(7):1057–78. doi: 10.1017/S0033291718003823 (PMC6498789; doi:10.1017/S0033291718003823)
Supplement: Supplementary file 1 [file S0033291718003823sup001.docx]

Appendix A

*Figure B1a. Forest plot of the association between sexual abuse and suicide attempts. Note random effects model used. OR = Odds ratio.*

*Figure B1b. Forest plot of the association between physical abuse and suicide attempts. Note random effects model used. OR = Odds ratio.*

*Figure B1c. Forest plot of the association between emotional abuse and suicide attempts. Note random effects model used. OR = Odds ratio.*

*Figure B1d. Forest plot of the association between any child abuse and suicide attempts. Note random effects model used. OR = Odds ratio.*

*Figure B1e. Forest plot of the association between emotional neglect and suicide attempts. Note fixed effects model used. OR = Odds ratio.*

*Figure B1f. Forest plot of the association between physical neglect and suicide attempts. Note random effects model used. OR = Odds ratio.*

*Figure B1g. Forest plot of the association between complex (repetitive incidents) abuse and suicide attempts. Note random effects model used. OR = Odds ratio.*

*Figure B2a. Forest plot of the association between sexual abuse and suicidal ideation. Note random effects model used. OR = Odds ratio.*

F

D

E

*Figure B2b. Forest plot of the association between physical abuse and suicidal ideation. Note random effects model used. OR = Odds ratio.*

*Figure B2c. Forest plot of the association between emotional abuse and suicidal ideation. Note random effects model used. OR = Odds ratio.*

*Figure B2d. Forest plot of the association between any child abuse and suicidal ideation. Note random effects model used. OR = Odds ratio.*

*Figure B2e. Forest plot of the association between emotional neglect and suicidal ideation. Note random effects model used. OR = Odds ratio.*

*Figure B2f. Forest plot of the association between physical neglect and suicidal ideation. Note random effects model used. OR = Odds ratio.*
